# Decreased vitamin D increase the risk for subclinical hypothyroidism in individuals with T2DM: a cross-sectional study

**DOI:** 10.3389/fnut.2025.1509465

**Published:** 2025-01-22

**Authors:** Yaling Fang, Xin Wen, Hui You, Yueye Huang, Shen Qu, Xingchun Wang, Le Bu

**Affiliations:** ^1^Department of Endocrinology and Metabolism, Shanghai Tenth People's Hospital, Tongji University School of Medicine, Shanghai, China; ^2^School of Medicine, Anhui University of Science and Technology, Huainan, China

**Keywords:** vitamin D, thyroid function, type 2 diabetes mellitus, subclinical hypothyroidism, obesity

## Abstract

**Background:**

Vitamin D is crucial for regulating calcium and phosphorus metabolism. More studies have revealed its role in chronic diseases. Our study aimed to examine the relationship between thyroid function and Type 2 Diabetes Mellitus (T2DM).

**Methods:**

730 patients with T2DM were enrolled in this cross-sectional study. Among them, 118 subjects were classified as obese, while 613 were classified as non-obese. Thyroid and 25 hydroxyvitamin D(25(OH)D) levels were measured. Patients were categorized into lower and higher VD groups based on the median. Thyroid function was compared between groups and their association was analyzed.

**Results:**

Body mass index (BMI), total cholesterol (TCH), triglyceride (TG), and free fatty acid (FFA) were significantly lower in the higher VD group compared to the lower VD group (all *p* < 0.05). In the higher VD group, free triiodothyronine (FT3) levels were significantly elevated (4.45 ± 0.93 vs. 4.95 ± 1.52 ng/mL, *p* < 0.001), while total triiodothyronine (TT4) (104.84 ± 21.17 vs. 99.99 ± 23.64 ng/mL, *p* = 0.008) and thyroid stimulating hormone (TSH) (2.88 ± 7.03 vs. 2.06 ± 1.72 ng/mL, *p* = 0.046) levels were significantly reduced compared to the lower VD group. VD showed a significant negative correlation with BMI, Glycosylated Hemoglobin (HbA1C), low-density lipoprotein (LDL-C), and FFA (*r* = −0.093, *p* = 0.016; *r* = −0.082, *p* = 0.036; *r* = −0.099, *p* = 0.011; *r* = −0.125, *p* = 0.001). FT3 and FT4 showed significant positive correlations with VD (*r* = 0.248, *p* < 0.001; *r* = 0.086, *p* = 0.025), while TT4 and TSH exhibited significant negative correlations (*r* = −0.103, *p* = 0.011; *r* = −0.080, *p* = 0.033). After adjusting for height, BMI, HGB, TCH, TG, FFA, and LDL, FT3 and FT4 remained significantly positively associated with VD (*r* = 0.227, *p* < 0.001; *r* = 0.089, *p* = 0.030), while TT4 and TSH continued to show significant negative associations (*r* = 0.091, *p* = 0.033; *r* = −0.081, *p* = 0.049). Linear regression analysis revealed a significant positive association between VD and FT3 (*β* = 4.144, *p* < 0.001) and negative associations with TT4 (*β* = −0.167, *p* < 0.001) and TSH (*β* = −0.412, *p* = 0.020). Logistic regression analysis indicated that VD serves as a protective factor against subclinical hypothyroidism (SCH) (OR 0.987, 95% CI 0.974–0.999, *p* = 0.035), even after adjusting for BMI, FBG, FINS, TCH, and HDL (OR 0.986, 95% CI 0.974–0.999, *p* = 0.041). T2DM patients with SCH had lower 25(OH)D levels compared to those without SCH (46.45 ± 4.76 vs. 45.40 ± 5.84 ng/mL, *p* = 0.029).

**Conclusion:**

These results suggest a dual relationship between VD and thyroid function. T2DM patients with SCH exhibited reduced VD levels.

## Introduction

Vitamin D (VD), a crucial fat-soluble vitamin, is abundantly found in various foods and is synthesized in the skin through exposure to sunlight. Its established role in maintaining bone health is primarily attributed to its ability to enhance the absorption of calcium and phosphorus, thereby supporting normal bone development and mineralization (1, 2). Furthermore, recent studies have suggested that VD extends beyond the maintenance of bone health, with its potential roles in various non-skeletal diseases garnering increasing attention (3). Increasing VD levels is believed to reduce the risk of various chronic diseases, including certain types of cancer, autoimmune diseases, atherosclerosis, and type 2 diabetes (T2DM). Research indicates that VD plays a crucial role in cell proliferation, differentiation, and apoptosis, which significantly impacts the occurrence and progression of cancer. For instance, studies have found that VD can lower the risk of breast, colorectal, and prostate cancers by inhibiting tumor cell proliferation and promoting the apoptosis of cancer cells. In the context of autoimmune diseases, VD is recognized for its role in modulating the immune system and reducing the incidence of autoimmune responses. It may play a significant role in the prevention of conditions such as multiple sclerosis and systemic lupus erythematosus by influencing T cell functionality and suppressing inflammatory responses. Additionally, VD deficiency has been closely linked to the progression of atherosclerosis. Research indicates that VD can enhance endothelial function and lower the risk of atherosclerosis, thereby exerting a beneficial effect on cardiovascular health (3–5).

In contemporary society, VD deficiency has emerged as a global health concern, significantly impacting overall health status. Research has demonstrated a strong association between VD deficiency and the incidence of various chronic diseases, particularly a markedly increased risk of T2DM. Epidemiological studies indicate a positive correlation between low VD levels and the prevalence of T2DM, with individuals deficient in VD facing a heightened risk of developing the disease (6). Further research has demonstrated that VD supplementation can effectively reduce the risk of T2DM and significantly enhance the rate of conversion from prediabetes to normal glucose levels. The role of VD in various biological processes, particularly in the regulation of insulin sensitivity and inflammatory responses, has received substantial attention. Specifically, VD is believed to improve insulin resistance by reducing systemic inflammation, a mechanism that is considered a critical pathway for its impact on T2DM (7). In addition, VD plays a crucial role in the regulation of glucose and lipid metabolism by enhancing muscle cell sensitivity to insulin, thereby increasing their capacity for glucose uptake. This mechanism suggests that VD may be central to the pathogenesis of metabolic syndrome and diabetes. Insufficient levels of VD can lead to a state of chronic inflammation, which not only adversely affects insulin secretion and action but may also disrupt lipid metabolism, thereby exacerbating the progression of diabetes (6).

VD plays a vital physiological role in the regulation of metabolic homeostasis. An expanding body of research evidence demonstrates a significant association between VD deficiency and metabolic syndrome (8). Vitamin D receptors (VDR) are expressed in immune cells, where they regulate proliferation and differentiation, thereby influencing the development of autoimmune diseases, including autoimmune thyroid disorders. VD deficiency has been recognized as a potential risk factor for these conditions, particularly for Hashimoto’s thyroiditis (HT) (9, 10). A case–control study indicated that for every 5 nmol/L increase in serum 25(OH)D levels, the risk of HT was reduced by a factor of 1.62 (11). Serum levels of 25(OH)D in patients with HT were significantly lower than those in the control group, suggesting that 25(OH)D may play a role in the progression from HT to hypothyroidism (12). In Chinese adults, VD insufficiency and deficiency are widespread, and low serum levels of 25(OH)D in women are associated with positive thyroglobulin antibodies (TgAb) (13).

Additionally, research has demonstrated a significant association between thyroid dysfunction and T2DM (14). Additionally, research has demonstrated a significant association between thyroid dysfunction and T2DM (15). Elevated levels of thyroid hormones are significantly associated with an increased risk of developing T2DM (16). The prevalence of both subclinical and overt hypothyroidism is notably higher in patients with T2DM (17). Specifically, among patients with T2DM, the prevalence of subclinical hypothyroidism (SCH) is 8.6%. This condition is characterized by elevated serum thyroid-stimulating hormone (TSH) levels while serum free T4 levels remain within the normal range (17). There exists a reciprocal relationship between diabetes and thyroid disease (18).

However, factors related to the changed thyroid function in T2DM have not been fully expounded. This study will investigate the association between VD and thyroid hormone in T2DM to verify their relationship.

## Materials and methods

### Subjects

**Figure 1:**
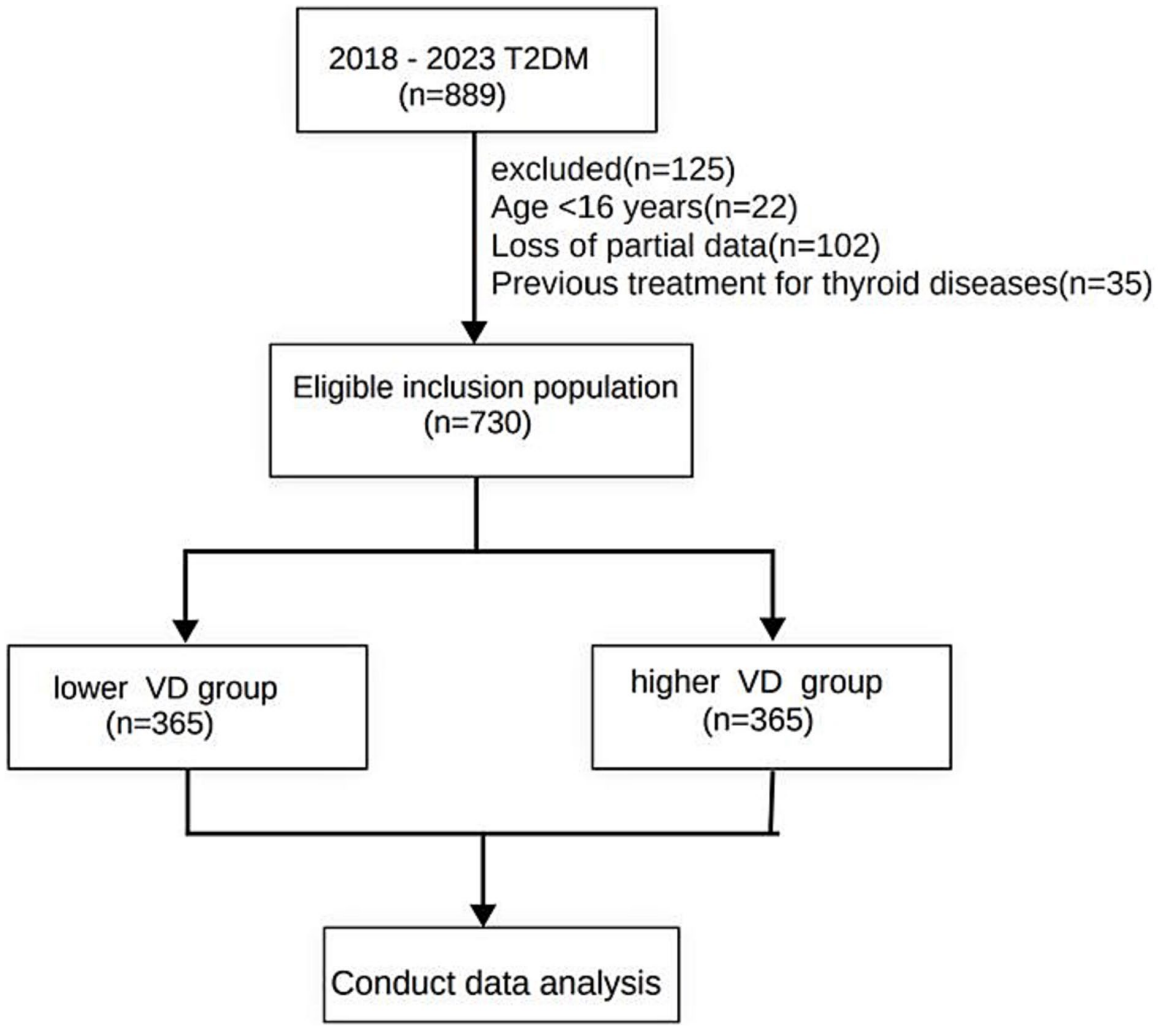
Flowchart of grouping and enrollment in this study.

A cross-sectional study included 889 T2DM patients treated at Shanghai Tenth People’s Hospital from July 2018 to August 2023. Following the exclusion criteria, 730 patients were included in the analysis, among them, 118 subjects were classified as obese, while 613 were classified as non-obese, as shown in Figure 1. Exclusion criteria included: (1) Age under 16 or over 75; (2) Prior thyroid-related surgery; (3) Autoimmune diseases or malignancies; (4) Mental illness; (5) Incomplete clinical data; and (6) Medications or treatments affecting thyroid hormones. In this study, all patients were categorized into two groups, namely the lower VD group and the higher VD group, based on the median value. The study has been approved by the Ethics Committee of Shanghai 10th People’s Hospital (approval no. 2012-RES-05).

Clinical trial number (NCT04548232) registration date is on October 9, 2022 registered in https://register.clinicaltrials.gov/.

### Measurements

A healthcare professional calculates the patient’s BMI using their height and weight measurements. The patient’s systolic and diastolic blood pressure, along with heart rate, were measured accurately after a ten-minute rest. All patients underwent an overnight fasting period, followed by blood tests at 8 am the next day. The measurement indicators included 25(OH)D; Glucose metabolism index: fasting plasma glucose (FPG), fasting insulin (FINS), Glycosylated Hemoglobin (HbA1C); lipid metabolism: total cholesterol (TCH), triglyceride (TG), high-density lipoprotein (HDL-C), low-density lipoprotein (LDL-C), free fatty acid (FFA), Thyroid function index: thyroid stimulating hormone (TSH), free triiodothyronine (FT3), free thyroxine (FT4), total thyroxine (TT3), and total triiodothyronine (TT4).

### Diagnostic criteria

Type 2 diabetes is diagnosed by the presence of classic symptoms (excessive thirst, frequent urination, increased hunger, unexplained weight loss) with a random blood glucose level ≥ 11.1 mmoL/L, a fasting blood glucose level ≥ 7.0 mmoL/L, a 2 h glucose tolerance test result ≥11.1 mmoL/L, or an HbA1C level ≥ 6.5% (19).

SCH is a condition characterized by having free T4 concentrations within the reference range, which indicates that the levels of thyroid hormone are adequate. However, TSH concentrations are above the reference range, suggesting that there is insufficient production of thyroid hormone (20).

In this particular study, we have utilized the standard set by China, which defines obesity as having a BMI equal to or greater than 28 kg/m^2^ (21).

### Statistical analysis

Data analysis was conducted using SPSS software (version 25). Continuous variables were reported as mean ± standard deviation (SD) or median (interquartile range, IQR), depending on data distribution, and categorical variables were presented as numerical values. The correlation between VD and other variables was analyzed using Pearson or Spearman methods, controlling for height, BMI, HbA1C, TCH, TG, FFA, and LDL-C. Variables significantly correlated at *p* < 0.05 were included in a multiple linear regression analysis to identify independent influencing factors. Binary logistic regression was employed to ascertain the independent predictors of VD level and SCH, without any adjustments made for the factors in model 1. Model 2 was adjusted for BMI, FBG, FINS, TCH, and HDL-C to address potential confounding factors. We illustrated 25(OH)D levels in T2DM patients with or without SCH and in patients with or without obesity using bar graphs. *p* < 0.05 indicates statistical difference.

## Results

### Baseline clinical characteristics

Table 1 demonstrates the anthropometric, metabolic, biochemical and hormonal characteristics of T2DM patients according to VD levels. A total of 730 patients with T2DM were included in this study, which were categorized into two groups based on serum VD levels: a low VD group (VD < 30.97 ng/mL) and a high VD group (VD ≥ 39.75 ng/mL), each containing 365 individuals. By comparing the physiological and biochemical indices of the two groups of patients, we found that there were significant differences in several parameters between the high VD group and the low VD group (*p* < 0.05). Specifically, BMI, TCH, TG, and FFA levels were significantly lower in the high VD group than in the low VD group (*p* < 0.05), suggesting that increased VD levels may be associated with improved metabolic health status. In addition, in terms of hormone levels, FT3 levels were significantly higher in the high VD group than in the low VD group, while TT4 and TSH levels were significantly lower. Specific data showed that the FT3 level in the high VD group was 4.45 ± 0.93 pmoL/L, whereas in the low VD group it was 4.95 ± 1.52 pmoL/L, a statistically significant difference (*p* < 0.001). Similarly, TT4 levels were 104.84 ± 21.17 nmoL/L in the high VD group compared to 99.99 ± 23.64 nmoL/L in the low VD group, and the difference was also significant (*p* = 0.008). TSH levels were 2.88 ± 7.03 mU/L in the high VD group compared to 2.06 ± 1.72 mU/L in the low VD group, and the difference was equally significant (*p* = 0.046).

**Table 1:** Anthropometric, metabolic, biochemical, and hormonal characteristics of patients with T2DM stratified according to VD.

Variables	VD < 30.97 (*n* = 365)	VD ≥ 39.75 (365)	*p* value
Age, years old	61.64 ± 13.04	61.47 ± 11.05	0.185
Gender, male/female	201/152	217/129	0.292
Height, m	1.65.39 ± 8.50	1.66.47 ± 8.10	0.093
Weight, kg	69.20 ± 14.90	68.17 ± 11.92	0.324
HR, beats/min	76.23 ± 12.54	73.02 ± 12.82	0.001**
BMI, kg/m^2^	25.21 ± 4.71	24.54 ± 3.62	0.039*
FPG, mmol/l	8.46 ± 3.49	8.26 ± 3.45	0.439
FINS, mU/L	30.50 (25.30, 34.80)	53.80 (46.95, 63.90)	0.416
HbA1c, %	9.17 ± 2.18	8.96 ± 2.05	0.203
TCH, mmol/l	4.49 ± 1.17	4.27 ± 1.06	0.014*
TG, mmol/l	1.99 ± 1.69	1.68 ± 1.37	0.009**
LDL-C, mmol/l	2.66 ± 1.04	2.54 ± 0.93	0.101
HDL-C, mmol/l	1.12 ± 0.34	1.15 ± 0.35	0.372
FFA, mmol/l	0.57 ± 0.26	0.52 ± 0.24	0.023*
FT3, pmol/l	4.45 ± 0.93	4.95 ± 1.52	<0.001***
FT4, pmol/l	17.17 ± 2.43	17.77 ± 8.24	0.193
TT3, nmol/l	1.58 ± 0.36	1.56 ± 0.55	0.407
TT4, nmol/l	104.84 ± 21.17	99.99 ± 23.64	0.008**
TSH, mU/l	2.88 ± 7.03	2.06 ± 1.72	0.046*

HR, heart rate; BMI, body mass index; FPG, fasting plasma glucose; FINS, fasting insulin; GHB, glycated hemoglobin; TCH, total cholesterol; TG, triglyceride; LDL-C, low-density lipoprotein; HDL, high-density lipoprotein; FFA, free fatty acid; FT3, free triiodothyronine; FT4 free thyroxine; TT3, total thyroxine; TT4, total triiodothyronine (TT4); TSH, thyroid stimulating hormone; * Statistically significant (*p* < 0.05). ** Statistically significant (*p* < 0.01). *** Statistically significant (*p* < 0.001).

### Correlation analysis

Table 2 demonstrates the analysis of the relationship between serum VD levels and other characteristics of all subjects. The results of the study showed that VD levels were significantly negatively correlated with BMI, HGB, LDL-C and FFA. Among them, the correlation coefficient between BMI and VD was *r* = −0.093 (*p* = 0.016), suggesting that higher BMI may be associated with lower levels of VD. HGB levels showed a similar trend with a correlation coefficient of *r* = −0.082 (*p* = 0.036), suggesting that anemia or altered blood-related indices may be associated with VD deficiency. In addition, the correlation coefficients between LDL-C and FFA were *r* = −0.099 (*p* = 0.011) and *r* = −0.125 (*p* = 0.001), respectively, further emphasizing the negative correlation between VD and lipid metabolism.

**Table 2:** Correlation between serum VD levels and other characteristics of all subjects.

Variables	All subjects (*n* = 730)	Adjusted for height, BMI, HGB, TCH, TG, FFA and LDL
	r(P)	r(P)
Age, years old	ns	/
Height, m	ns	/
Weight, kg	ns	/
BMI, kg/m^2^	−0.093 (0.016)*	/
HR, beats/min	−0.145 (<0.001)***	/
SBP, mmHg	ns	/
DBP, mmHg	ns	/
FPG, mmol/l	ns	/
FINS, mU/L	ns	/
HbA1c, %	−0.082 (0.036)*	/
TCH, mmol/l	−0.141 (<0.001)	/
TG, mmol/l	−0.168 (<0.001)	/
HDL-C, mmol/l	ns	/
LDL-C, mmol/l	−0.099 (0.011)*	/
FFA, mmol/l	−0.125 (0.001)**	/
FT3, pmol/l	0.248 (<0.001)***	0.227 (<0.001)***
FT4, pmol/l	0.086 (0.025)*	0.089 (0.030)*
TT3, nmol/l	ns	/
TT4, nmol/l	−0.103 (0.011)*	−0.091 (0.033)*
TSH, mU/L	−0.080 (0.033)*	−0.081 (0.049)*

HR, heart rate; BMI, body mass index; SBP, systolic blood pressure; DBP, diastolic blood pressure; FPG, fasting plasma glucose; FINS, fasting insulin; GHB, glycated hemoglobin; TCH, total cholesterol; TG, triglyceride; HDL, high-density lipoprotein; LDL-C, low-density lipoprotein; FFA, free fatty acid; FT3, free triiodothyronine; FT4 free thyroxine; TT3, total thyroxine; TT4, total triiodothyronine (TT4); TSH, thyroid stimulating hormone. * Statistically significant (*p* < 0.05). ** Statistically significant (*p* < 0.01). *** Statistically significant (*p* < 0.001). NS, no significant.

On the other hand, FT3 and FT4 were significantly positively correlated with VD levels, with correlation coefficients of *r* = 0.248 (*p* < 0.001) and *r* = 0.086 (*p* = 0.025), respectively, suggesting that VD may enhance thyroid hormone secretion by boosting thyroid function, which may then positively affect metabolic processes. In contrast, TT4 and TSH were significantly negatively correlated with VD levels, with a correlation coefficient of *r* = −0.103 (*p* = 0.011) for TT4 and *r* = −0.080 (*p* = 0.033) for TSH, suggesting that high VD levels may inhibit TSH secretion and thus improve thyroid function.

After adjusting for height, BMI, HGB, TCH, TG, FFA and LDL, FT3 and FT4 were still significantly positively correlated with VD, with correlation coefficients *r* = 0.227 (*p* < 0.001) and *r* = 0.089 (*p* = 0.030), respectively. Also, TT4 and TSH continued to show negative correlation with VD, with correlation coefficients of *r* = −0.091 (*p* = 0.033) for TT4 and *r* = −0.081 (*p* = 0.049) for TSH. These results suggest that VD is not only significantly associated with metabolic profiles, but may also influence thyroid function and endocrine regulatory mechanisms.

### Independent influencing factors of thyroid function

In order to further investigate the effect of VD level and its confounding factors on thyroid function, linear regression analysis was performed on all subjects in this study, and the results are shown in Table 3. The analysis showed a significant positive correlation between VD level and FT3 with a regression coefficient of *β* = 4.144 (*p* < 0.001), which suggests that an increase in VD may be closely related to an increase in FT3 level. This finding suggests that VD may play an active role in metabolic regulation by regulating thyroid function and promoting FT3 synthesis or secretion.

**Table 3:** Liner regression analysis of VD and thyroid function.

Variables	*β*	95%CI	*p* value
BMI, kg/m^2^	−0.152	(−0.472,0.168)	0.352
HR, beats/min	−0.195	(−0.304, −0.085)	0.001**
HbA1c, %	0.089	(−0.569, 0.747)	0.791
TCH, mmol/l	−0.404	(−3.870, 3.062)	0.819
TG, mmol/l	−1.304	(−2.517, −0.091)	0.035*
LDL-C, mmol/l	−0.541	(−4.181, 3.099)	0.771
FFA, mmol/l	−2.173	(−7.626, 3.281)	0.434
FT3,pmol/l	4.144	(2.996, 5.292)	<0.001***
FT4, pmol/l	0.085	(−0.128, 0.299)	0.431
TT4, nmol/l	−0.167	(−2.231, −0.102)	<0.001***
TSH, mU/L	−0.412	(−0.758, −0.066)	0.020*

HR, heart rate; BMI, body mass index; FPG, fasting plasma glucose; GHB, glycated hemoglobin; TCH, total cholesterol; TG, triglyceride; HDL, high-density lipoprotein; LDL-C, low-density lipoprotein; FFA, free fatty acid; FT3, free triiodothyronine; FT4 free thyroxine; TT3, total thyroxine; TT4, total triiodothyronine (TT4); TSH, thyroid stimulating hormone; * Statistically significant (*p* < 0.05). ** Statistically significant (*p* < 0.01). *** Statistically significant (*p* < 0.001). CI, confidence interval.

On the other hand, linear regression analysis also revealed a significant negative correlation between VD levels and TT4 and TSH. the regression coefficient for TT4 was *β* = −0.167 (*p* = 0.001), while that for TSH was *β* = −0.412 (*p* = 0.020). This suggests that higher VD levels may be associated with lower TT4 and TSH, further supporting the potential role of VD in regulating thyroid function.

### Independent influencing factors of SCH

In this study, 132 patients were diagnosed with SCH, while 598 patients were not affected by SCH. To assess the correlation between VD levels and SCH, logistic regression analysis was performed on all participants in this study, and the results are detailed in Table 4. The results of logistic regression analysis showed that VD level was a significant protective factor for SCH, OR = 0.987, (95% CI: 0.974–0.999, *p* = 0.035). This result suggests that increased levels of VD may be associated with a reduced risk of developing SCH.

**Table 4:** Logistic regression analysis of VD and SCH.

	Variables	OR (95%CI)	*p* value
Model 1	VD	0.987 (0.974,0.999)	0.035*
Model 2	VD	0.986 (0.974,0.999)	0.041*
	BMI	1.011 (0.962, 1.062)	0.666
	FBG	1.017 (0.957, 1.080)	0.578
	FINS	1.001 (0.999, 1.002)	0.462
	TCH	1.067 (0.882, 1.291)	0.501
	HDL-C	1.388 (0.761, 2.531)	0.285

Model 1, unadjusted; Model 2, adjusted for BMI, FBG, FINS, TCH, HDL. VD, vitamin D; BMI, body mass index; FPG, fasting plasma glucose; FINS, fasting insulin; TCH, total cholesterol; HDL, high-density lipoprotein; OR odds ratio, CI confidence interval. *Statistically significant (*p* < 0.05).

The association remained significant after further controlling for confounders, including BMI, FBG, FINS, TCH, and HDL-C, with an adjusted OR = 0.986, (95% CI: 0.974–0.999, *p* = 0.041). This suggests that the negative correlation between VD levels and SCH remains significant even after accounting for these potential confounders, emphasizing the important role of VD in thyroid health.

In this study, we performed a stratified analysis of patients with T2DM to compare differences in 25(OH)D levels in different subgroups, with a special focus on patients with concomitant SCH and obesity. The results showed that T2DM patients with SCH had significantly lower 25(OH)D levels than those without SCH, with specific values of 46.45 ± 4.76 nmol/L versus 45.40 ± 5.84 nmol/L, and the difference was statistically significant (*p* = 0.029). This finding suggests that SCH may be associated with VD deficiency, which may affect metabolic status and management of related diseases.

Furthermore, in the comparison between the obese and non-obese groups, the results showed that the 25(OH)D levels in the obese group were significantly lower than those in the non-obese group, 39.86 ± 16.82 nmol/L versus 47.75 ± 17.76 nmol/L, respectively, and the difference was also statistically significant (*p* = 0.016). This result suggests that obesity may be an important factor in the reduction of VD levels, suggesting that VD monitoring and intervention should not be neglected in the clinical management of obese patients.

**Figure 2:**
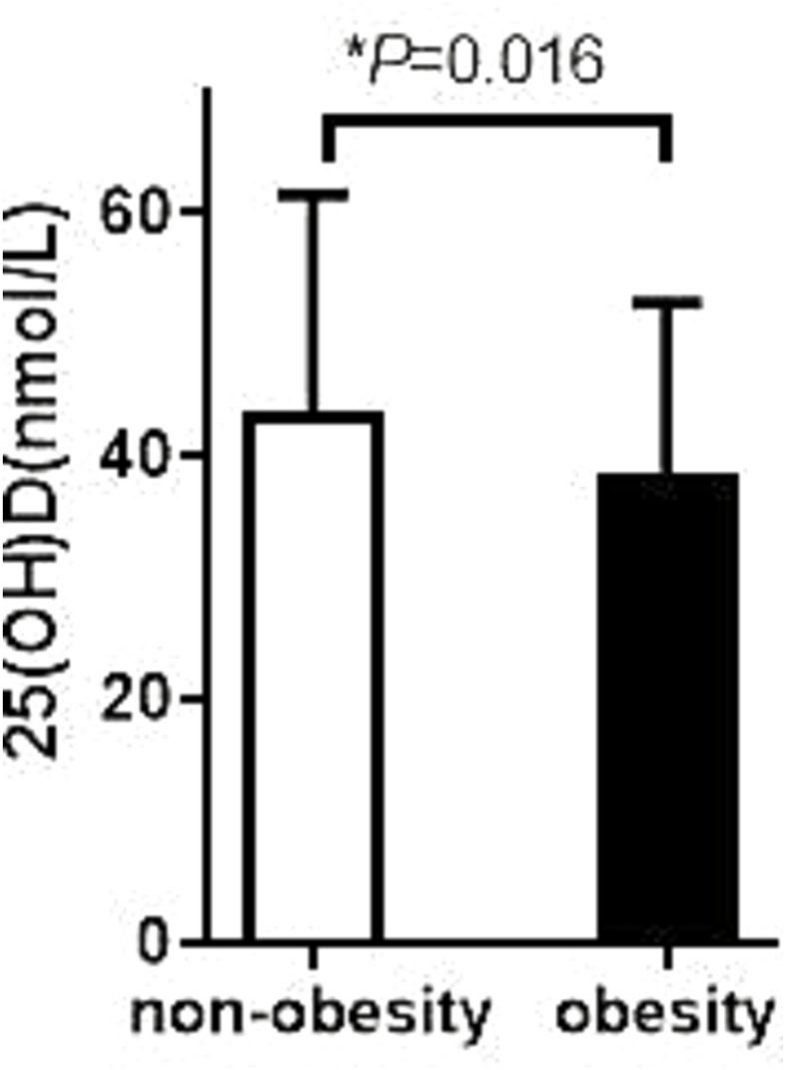
Comparison of VD levels between obese patients with or without SCH. The 25 (OH) D level in the non-SCH group was 46.45 ± 4.76 ng/mL; The 25 (OH) D level in the SCH group was 46.45 ± 4.76 ng/mL; *p* = 0.029.

**Figure 3:**
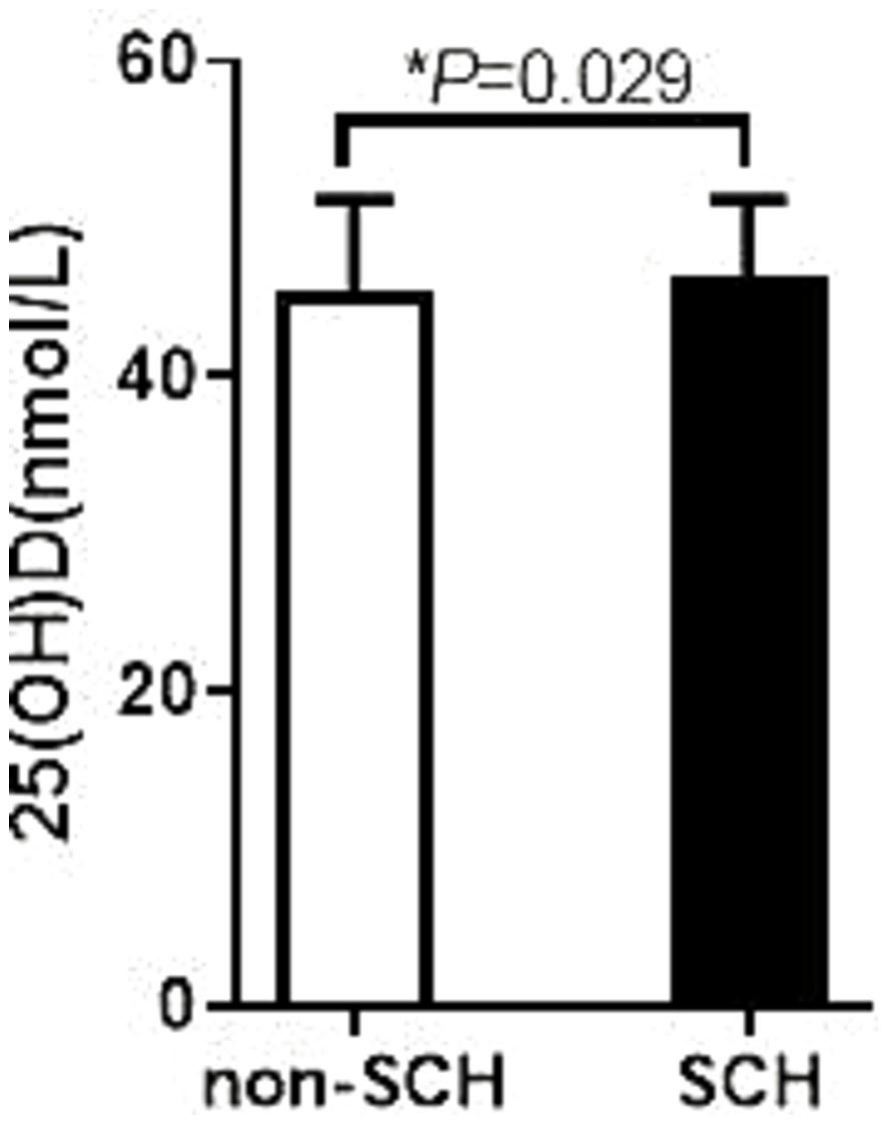
Comparison of thyroid hormone among obese patients with different degree of VD. The 25 (OH) D level in the non-obesity group was 47.75 ± 17.76 ng/mL; The 25 (OH) D level in the obesity group was 39.86 ± 16.82 ng/mL; *p* = 0.016.

What’s more, the analysis also found a negative correlation between VD levels and thyroid function in T2DM patients. This correlation was particularly evident in T2DM patients with SCH or obesity, suggesting that VD levels are generally low in these patient populations (see Figures 2, 3 for details). This may reflect the important role of VD in regulating thyroid function and metabolic health. Additionally, glucose level (FBG and HbA1c) had no difference between SCH and non-SCH group (8.62 ± 3.53 vs. 8.34 ± 3.53 mmoL/L, *p* = 0.446, 8.8 ± 1.92 vs.9.18 ± 2.19%, *p* = 0.099), as shown in Table 5.

**Table 5:** T2DM glucose levels in SCH and non-SCH groups.

Variables	SCH Group (*n* = 132)	Non-SCH Group (*n* = 598)	*p*-value
FPG (mg/dL)	8.62 ± 3.53	8.34 ± 3.53	0.446
HbA1c (%)	8.80 ± 1.92	9.18 ± 2.19	0.099

FPG, fasting plasma glucose; HbA1c, glycated hemoglobin.

## Discussion

T2DM as a worldwide health issue, not only increase the risks for diabetic complications but also associated with poor nutrition (22). T2DM is associated with glucose and lipid metabolism disorders and vitamin D deficiency (23). The nutrition disorders including VD deficiency in diabetes may also has influence on other metabolic disorders in T2DM (24).

Multiple cross-sectional studies have shown a notably higher prevalence of thyroid disorders in patients with T2DM (17, 25, 26). A large study revealed that thyroid dysfunction prevalence in men with T2DM is 6.9, and 10.9% in women. Hypothyroidism in individuals results in reduced glucose utilization by peripheral tissues, causing slower glucose oxidation and synthesis. Consequently, insulin is unable to effectively maintain the utilization of glucose by muscles, resulting in the occurrence of insulin resistance in patients (27, 28).

In SCH, serum TSH levels are typically elevated, while T3 and T4 levels remain within the normal range, indicating that the thyroid gland is still capable of maintaining some degree of hormonal secretion. However, as the condition progresses, a further decline in thyroid function may lead to a gradual decrease in T3 and T4 levels, potentially advancing to overt hypothyroidism. Consequently, elevated TSH serves as a key diagnostic marker for SCH, reflecting a mild impairment of thyroid function, whereas the preservation of normal T3 and T4 levels suggests that thyroid function has not yet reached a state of overt dysfunction. Monitoring TSH levels is therefore of critical clinical importance in the diagnosis and management of SCH, particularly for the early detection and intervention of disease progression.

Numerous studies have explored the role of VD in thyroid diseases, but a definitive consensus remains elusive. This study examines the correlation between VD levels and thyroid function in T2DM patients. Our study showed a significant association between VD levels and thyroid hormone levels in patients with T2DM. VD showed a positive correlation with FT3 and a negative correlation with TT4 and TSH. After adjusting for height, BMI, HbA1C, TCH, TG, FFA, and LDL-C, FT3 and FT4 remained significantly positively associated with vitamin D levels. TT4 and TSH remained significantly negatively correlated with vitamin D levels. Prior research in this area has been controversial.

The phenomenon of TT4 being lower than FT4 at higher vitamin D levels can be explained by the following mechanisms: (1) Thyroid Hormone Metabolism Regulation: Vitamin D may promote the conversion of T4 to its active form, FT4, thereby increasing FT4 concentration without significantly affecting TT4 levels (29). (2) TSH Regulation: Higher vitamin D levels may reduce TSH secretion, decreasing the demand for thyroid hormone synthesis, leading to an increase in FT4 while keeping TT4 at lower levels (30). (3) TBG Influence: Vitamin D may regulate the synthesis of thyroid hormone-binding globulin (TBG), reducing TT4 levels and maintaining higher FT4 concentration (31). (4) Immune Modulation: Vitamin D may reduce thyroid inflammation, promoting the production of FT4 and inhibiting TT4 synthesis. In summary, the phenomenon of TT4 being lower than FT4 at higher vitamin D levels involves multiple mechanisms, including thyroid hormone metabolism, immune modulation, and TSH feedback. Future research should further explore the interactions of these mechanisms.

Sandeep et al. observed a positive correlation between 25(OH)D levels and FT3 and FT4 levels (32). Additionally, they identified a negative correlation between 25(OH)D and TSH levels. These findings align with our results, demonstrating a comparable relationship between vitamin D and thyroid function. However, the study conducted by Ke et al. Contrary findings indicated no link between vitamin D deficiency and TSH or FT4 levels in Hashimoto’s thyroiditis patients (33). The significant difference in findings may be due to not categorizing patients with Hashimoto’s thyroiditis or hypothyroidism by their 25(OH)D levels All of the cases included in our study involved patients with T2DM, and among them, some patients were obese. Obesity may have influenced the experimental outcomes.

Previous studies have demonstrated a significant association between SCH and an elevated risk of depression, along with declines in quality of life, cognitive function, and memory, compared to individuals with normal thyroid function. The incidence of this disease varies across different populations, typically ranging from 3 to 15%. Age, suboptimal iodine status, and being female are identified as high-risk factors associated with the development of this condition (34–36). Our study also investigates the association between VD and SCH. Logistic regression analysis shows that VD serves as a protective factor for SCH. This association remains significant after adjusting for BMI, FBG, FINS, TCH, and HDL-C.

We compared the 25(OH)D levels in T2DM patients with and without SCH. We analyzed 25(OH)D levels in T2DM patients, comparing those with obesity to those without. The study identified that T2DM patients with SCH exhibited lower 25(OH)D levels than those without SCH (46.45 ± 4.76 vs. 45.40 ± 5.84, *p* = 0.029). Obese patients with T2DM exhibited significantly lower levels of 25(OH)D than non-obese individuals. These findings provide important insights for further exploring the relationship between VD and thyroid function. Our study found a negative association between VD levels and thyroid function in T2DM patients. Additionally, our findings demonstrated that T2DM patients with SCH or obesity exhibited lower vitamin D levels compared to those without SCH or obesity.

One limitation of this study is its cross-sectional design, which cannot reveal causal relationships. Due to the lack of long-term follow-up data, this study was unable to assess the temporal changes in vitamin D levels and thyroid function in patients with type 2 diabetes (T2DM), nor their potential causal links. Therefore, future longitudinal studies will be helpful in further understanding the dynamic relationship between vitamin D and thyroid function in T2DM patients, as well as the underlying causal mechanisms.

Another limitation is that the study did not provide a detailed analysis of the medication use and dosage in T2DM patients. The differences in VD levels in T2DM patients are influenced not only by the medications used and their dosages but also by multiple factors such as lifestyle, metabolic status, and genetic background. As this study primarily aimed to explore the relationship between vitamin D and thyroid function in T2DM patients, it did not discuss in-depth the specific factors affecting vitamin D levels. Future research should further refine these factors to more comprehensively reveal the mechanisms behind the changes in vitamin D levels in T2DM patients and their interactions with thyroid function.

## Conclusion

In conclusion, our study revealed a dual relationship between VD levels and thyroid function in individuals diagnosed with T2DM, when vitamin D levels are higher, FT3 and FT4 concentrations are increase, while TT4 and TSH concentrations decrease. Notably, T2DM patients with SCH or obesity exhibited significantly lower VD levels compared to those without SCH or obesity. These findings underscore the need for further research into the relationship between vitamin D and thyroid function in this patient population.

## Data Availability

The original contributions presented in the study are included in the article/supplementary material, further inquiries can be directed to the corresponding authors.
